# Antibiotic drug-resistance as a complex system driven by socio-economic growth and antibiotic misuse

**DOI:** 10.1038/s41598-019-46078-y

**Published:** 2019-07-05

**Authors:** Bhawna Malik, Samit Bhattacharyya

**Affiliations:** Disease Modelling Lab, Department of Mathematics, School of Natural Sciences, Shiv Nadar University, Gautan Buddha Nagar, India

**Keywords:** Diseases, Computational biology and bioinformatics

## Abstract

Overwhelming antibiotic use poses a serious challenge today to the public-health policymakers worldwide. Many empirical studies pointed out this ever-increasing antibiotic consumption as primary driver of the community-acquired antibiotic drug-resistance, especially in the middle- and lower-income countries. The association is well documented across spatio-temporal gradients in many parts of the world, but there is rarely any study that emphasizes the mechanism of the association, which is important for combating drug-resistance. Formulating a mathematical model of emergence and transmission of drug-resistance, we in this paper, present how amalgamating three components: socio-economic growth, population ecology of infectious disease, and antibiotic misuse can instinctively incite proliferation of resistance in the society. We show that combined impact of economy, infections, and self-medication yield synergistic interactions through feedbacks on each other, presenting the emergence of drug-resistance as a self-reinforcing cycle in the population. Analysis of our model not only determines the threshold of antibiotic use beyond which the emergence of resistance may occur, but also characterizes how fast it develops depending on economic growth, and lack of education and awareness of the population. Our model illustrates that proper and timely government aid in population health can break the self-reinforcing process and reduce the burden of drug-resistance in the community.

## Introduction

The rapid emergence and dissemination of resistant bacteria is occurring globally, threatening the potency of antibiotics, which saves millions of lives^1,2^. It is described as the primary threat to public health in the 21st century, and agencies around the globe have underlined the urgent need for actions^3,4^. There is high resistance to Cotrimoxazole (68.5%) and Ampicillin (84.5%) among the Gram-negative bacilli in Zimbabwe^5^. In the same country, Gram-positive cocci is also resistant to Cotrimoxazole (69%) and Nalidixic acid (81%). *E. coli* is another pathogen that shows 84% resistance to ampicillin^6^. A recent report has documented the non-susceptibility pattern of key Gram-negative (such as *Klesbsiella, E. coli, Salmonella, Shigella*) and Gram-positive pathogens (such as *S. pneumoniae, S. aureus, S. agalactae, MRSA*) to the most common antibiotics in Sub-Saharan Africa^7^. However, antibiotic resistance is worldwide, and the effects of resistance are more severe in lower- and upper middle-income countries (Fig. S1)^8,9^. This ever-growing problem not only threatens public health, but also incurs a huge toll on nation’s economic growth by delayed hospitalizations, lengthening recovery time, expensive medicines, and specialized care for patients^10–12^. Shrestha *et al*. (2018) have estimated the economic cost of resistance per antibiotic by drug class and compared those in developing and developed countries, like in Thailand and United States^11^. Moreover, mortality and morbidity are the indirect consequences of resistance that incur a huge loss in income leading the sufferer and family to the poor economic condition^13^.

Antibiotic resistance may occur naturally, but irrational use of antibiotics accelerate the process leading to expulsion of sensitive strain, and procreation and propagation of the resistant bacteria in the community^11,14,15^. Several countries already have taken steps to reduce the misuse of antimicrobials. World Health Organization (WHO) already has launched a Global Action Plan in 2015 to initiate evidence based prescribing through effective, rapid, and low-cost diagnostic tools to optimize use of antimicrobials^16^. In spite of these efforts, the volume of antibiotic use is ever-increasing worldwide, especially in developing countries. A recent report indicates that the global antibiotic consumption increased by 65% (rate increased by 39%) between 2000 and 2015, from 21.1 to 34.8 billion Daily Defined Doses (DDDs), and the global increase was primarily driven by increased consumption in Lower Middle Income Countries (Fig. S2)^9^. Countries like Brazil, Russia, India, China, and South Africa (BRICS countries) are developing nations, which show the highest drug consumption from 2000 to 2010 with India as the first and China is in the second position^17^.

Self-medication plays a major role in ever-increasing antibiotic consumption^18–21^. Over-the-counter (OTC) sales – which is more ubiquitous in more economically destitute society – is one of the source of self-medication^22^. It is a very common practice in countries like Bangladesh^23^, Addis Ababa and Central Ethiopia^24^, and North West Ethiopia^25^. Avoiding expenditure of treatment, inability to access medical facilities, less education and lack of awareness are utmost challenges for self-medication^26^. Also, perceived patient expectation, lack of knowledge and diagnostics, incentives and advertising from industry, and financial benefits are major factors for irrational antibiotic prescription by physicians in most of the developing countries^27^.

The relation between antimicrobial use and emergence of resistance is a complex association. While most of the recent research focus on understanding microbiology of drug-resistance, there are only few modelling studies that emphasize transmission dynamics and coexistence of strains under volume of antibiotic use. For example, Massad *et al*. (1993) studied coexistence of sensitive and resistant strains in hospital infection^28^. Austin *et al*. (1999, 2001) developed model to understand influence of drug use on transmission of resistance^29^. In the past few years, various antibiotic drug-resistance models have been developed that discuss usage of antibiotics and emergence of resistance^30–34^. For example, Levin *et al*. (2014) have shown by developing simple epidemiological model that burden of resistance can be maintained under certain acceptable level if the use of specific antibiotics declines with the frequency of resistance to these drugs^35^. Developing game theoretic model, Fu and Chen (2018) have shown how social learning may help prescribing behavior of physicians to promote social optimum of antibiotic consumption^36^. However, there is rarely any modelling work that integrate socio-economic growth, antibiotic use and transmission dynamics to understand this ever-increasing antibiotic consumptions in middle and lower income countries.

**Figure 1:**
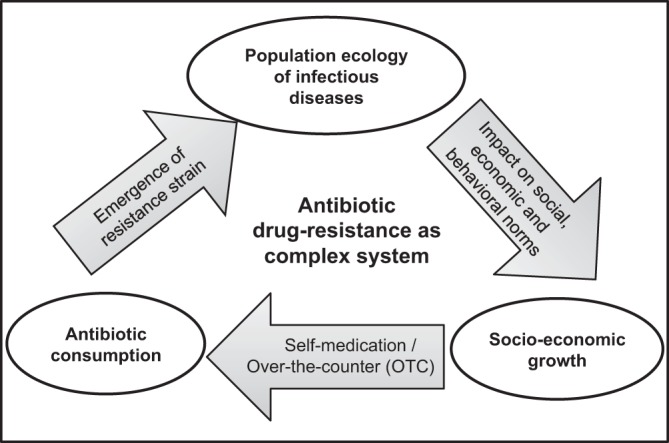
Antibiotic drug-resistance as an integrated system driven by population ecology of infectious disease, socio-economic growth of population and antibiotic consumption by individuals in the population.

In this paper, we present a mechanistic model of community-acquired drug-resistance, combining population ecology of infectious disease, economic growth and antibiotic use as a function of individual economic status. Every country has its own economic constraints and epidemiological parameters behind the emergence and prevalence of drug resistance in its population. Also, different pathogens have different mechanisms of emergence and transmission. Instead of incorporating specific characteristics of a diverse variety of pathogens, we test our theory using general susceptible-infected-susceptible (*SIS*) model, where individuals can have recurrent infections over the course of their lifetime. This kind of general framework of reinfection is considered to represent the repeated threat of infection that individuals face in most of developing tropical countries. However, our model can demonstrate the emergence of antibiotic drug-resistance in the population as a self-reinforcing system where these three components interact through positive feedbacks on each other (Fig. 1). It can explain the ever-increasing development of resistance, especially in lower-income countries. We interpret how invasion and dominance of resistance are correlated with socio-economic growth and antibiotic consumption in the population. Our model analyses reveal that a large inflow of capital in the form of development aid, especially in the early stage, can escape the situation by encouraging economic growth and scaling down the antibiotic misuse.

## Material and Methods

Our drug-resistance model connects three domains of human population: *socio-economic growth*, *ecology of infectious disease*, and *antibiotic (mis)use by individuals*. Although these three aspects are dynamic in nature and canonical in their respective disciplines, combining them may explain the evolution of drug-resistance in the population. Below, we describe model formulation in these three parts separately, and then develop the integrated model. Detailed model development of separate components are discussed in the Supplementary Information.

### Model of economic growth

We consider the model of economic growth based upon three driving forces namely, capital (including human capital), labor and technology. This is described as Neoclassical growth theory in economics that layouts how steady economic growth rate can be accomplished with these three forces^37–39^. The relationship between the labor and capital determines the output (i.e., the income), thus the model provides a basis for economic growth in the canonical framework by defining a process that describes capital conversion and accumulation in population over time^40^. This is a macro-oriented approach that dictate patterns of capital accumulation as an inevitable outcome of normal market processes driven by savings and technological progress. The patterns of accumulation converges over time leading to a steady economic growth across countries^40^.

We use a linear formulation of *Constant-elasiticity-of-substitution* (CES) production model, which is one of the massively used function in economic analysis^41^. Thus, the rate of change of capital is given by:

1 $$\frac{dh}{dt}=\{{r}_{h}h+{r}_{l}\}-{\delta }_{1}h(t),$$

where *h*(*t*) is the capital (income) at time *t* scaled to the labour supply. *r*_*h*_ and *r*_*l*_ determines the share in total output from capital and labor respectively, so *r*_*h*_ + *r*_*l*_ = 1. *δ*_1_ is the rate of capital depreciation. Detailed discussion and derivation of the linear form is given in the Supplementary Information (SI).

### Model of strain dynamics: antibiotic resistance

Acquired resistance may emerge from several biological mechanisms such as through mutation within the existing genome or through the plasmid transfer^42^. For instance, resistance to the rifampicin drug in *M.tuberculosis* emerges through mutation whereas resistance to antibiotic *β*-lactam in *E. coli* was by plasmid transfer^43^. However, the key issue in this epidemiological model framework is to analyze the role of antibiotic consumption in the onset and propagation of resistance in the population. We assume that the drug-resistant strain is already present and circulating in the population. For simplicity, we also assume that individual in population are either colonized by sensitive strains or by drug-resistant strains. Colonization may result in the morbidity, and people undergo treatment also. We also consider that a susceptible individual may even consume antibiotics without having any prior knowledge of underlined infection, but the drug has no effect if individual is colonized by resistant strain.

**Figure 2:**
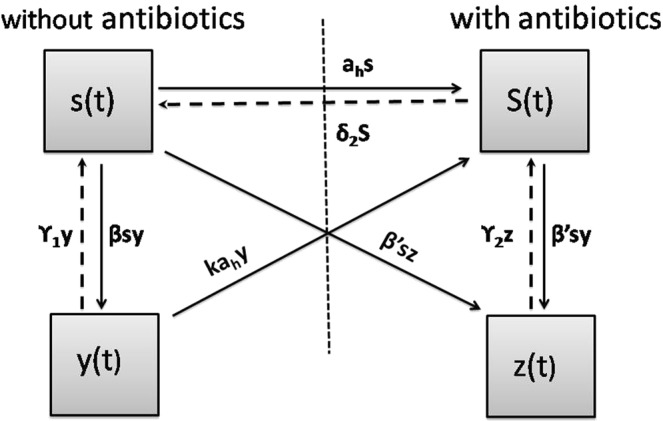
Schematic of the model. For detail explanation, see the Supplementary Information.

We consider a general *SIS* framework, where individuals become susceptible once recovered from an infection. Based on these assumptions, we compartmentalize the total population based on whether individuals are using antibiotics or not, and further based on individual infection status – by the sensitive or the drug-resistant strain. Figure 2 shows the schematic of the model and Table 1 provides description of the parameters used in the model. Detailed description of the model is given in the Supplementary Information.

**Table 1:** Description of variables and baseline parameter values (or ranges).

Parameters	Description	Values	Reference
$\hat{\alpha }$	maximum antibiotic use	3, (1–5)	calibrated
$\tilde{\alpha }$	minimum antibiotic use	0.01, (0.001–0.1)	calibrated
${r}_{h}^{c}$	intrinsic growth rate of capital	0.9	^56^
${r}_{l}^{c}$	per capita amount spend on training or education of labor	0.8	^56^
*h*_*o*_	education and awareness level	8, (7–15)	calibrated
*k*	proportion of individuals who are recovered due to drug use	0.1	calibrated
*p*	proportion of individuals who use antibiotics even after recovery	0.1	calibrated
*ρ*	proportion of infected (resistant) individuals take treatment	0.2	calibrated
*δ*_1_	rate of capital depreciation	0.1	^56^
*δ*_2_	average duration of antibiotic use	3/month	^29^
${ {\mathcal R} }_{o}$	reproduction number for sensitive strains	8, (7–9)	^29^
${ {\mathcal R} }_{o}^{\text{'}}$	reproduction number for drug-resistant strain	10, (9–11)	^29^
*γ*_1_	recovery rate of sensitive strains	0.4/month	^29^
*γ*_2_	recovery rate of drug-resistant strains	0.3/month	^29^
*c*_*z*_	per capita cost of treatment of severe infections	0.8 (0.1–2)	calibrated
*μ*	mortality rate	$\frac{1}{72}$/year	
**Variables**	**Description**		
*s*(*t*)	Susceptible		
*S*(*t*)	Susceptible using antibiotics		
*y*(*t*)	Infective with sensitive commensals		
*z*(*t*)	Infective with resistant commensals		
*h*(*t*)	Capital or income		

Transmission potential of the commensal bacteria is measured by basic reproduction number (${ {\mathcal R} }_{o}$), which is given by^38^:

2 $${ {\mathcal R} }_{o}=\frac{\beta }{\mu +{\gamma }_{1}}$$

In our model framework, resistant strains have a lower biological fitness as compared to the sensitive strains in absence of drug. Irrational and extensive use of antibiotics exert a selective pressure by killing sensitive bacteria and enable resistant bacteria to thrive and multiply^44^. The basic reproduction ${ {\mathcal R} }_{o}^{^{\prime} }$ for resistant strain is given by:

3 $${ {\mathcal R} }_{o}^{^{\prime} }=\frac{{\beta }^{^{\prime} }}{\mu +{\gamma }_{2}}$$

The dynamics of population infected by these two strains are determined by the parameters ${ {\mathcal R} }_{o}$ and ${ {\mathcal R} }_{o}^{^{\prime} }$. We shall assume that these are greater than unity (otherwise the commensal strains will never become established). According to the ecological theory, individual strain is established in the population when the basic reproduction rate is greater than one, but strain with maximum basic reproduction number will always dominate over the other strain. So here, the sensitive strain will dominate the population in absence of drug. Increased antibiotic consumption however, can disrupt the balance in their reproductive ratios such that it reduces the fitness cost of resistant strain and create a niche in the population of individuals treated with antibiotics.

### Coupled disease-economic growth model

In reality infectious disease and economic growth are strongly coupled^45–47^. The correspondence between income and health is collective and circular causation: income influences health and health influences income. Mechanism of infectious disease and economies are axiomatically allied as poor health shrinks the economy by decreasing the capital and labor productivity, while the economic wellness impacts the health like nutrition, sanitation, treatment, etc. Prevention and treatment of infections from drug-resistant strains also thrust a strong financial burden worldwide, especially on low- and middle-income countries by churning out huge sum of income or capital^48^.

We couple the two strains disease model and economic growth model by assuming that infections have detrimental impact on human capital accumulation and labor supply. It is based on recent literature studies in the field of epidemiology and economics that human health have direct impact on cognitive, physical, and social development which eventually have repercussion on economic development in future^49–51^. We consider that the share parameters *r*_*h*_ and *r*_*l*_ in the economic growth equation (1) are functions of strains prevalences as follows:

4 $${r}_{h}(y,z)={r}_{h}^{c}\mathrm{(1}-y\mathrm{)(1}-z)$$

5 $${r}_{l}(y,z)={r}_{l}^{c}\mathrm{(1}-y\mathrm{)(1}-z)$$

where parameters ${r}_{h}^{c} > 0$ and, ${r}_{l}^{c} > 0$ determine the growth of capital in absence of any infection in the population.

### Antibiotic misuse

Antibiotic consumptions in developing and underdeveloped countries are strongly correlated with the economic growth of the populations. Several studies pointed out that poor economy fosters self-medication practice in individuals, especially through over-the-counter (OTC) sales^18,19,22^. We develop a linear function of antibiotic use that depends on income on the basis of assumption that when income (capital) is low, then self-medication is high and reaches a maximum level $\hat{a}$. Similarly, when income is high then antibiotic use reaches a minimum level $\tilde{a}$. The function is given by:

6 $$a(h)=mh+\hat{a}$$

where slope of function *m* is determined by

7 $$m=\frac{\tilde{a}-\hat{a}}{{h}_{o}} < 0$$

The parameter *h*_*o*_ determines the effect of education and awareness of the community on antibiotic consumption. A schematic of the function is given in Fig. S3 in SI. Figure S3 shows that higher the *h*_*o*_, lower the level of awareness and hence higher the antibiotic consumption.

In reality, the association between the behavioral aspects of individual medical practice and socio-economic growth of population is more complex and nonlinear, and there is rarely any empirical contribution in support of this. Here, we intentionally consider a simpler approach, not to discount complexity, but to illustrate the complex outcomes as emergent properties of parsimonious integrated models.

### Integrated model and analysis

We integrate the three components and construct the conceptual framework that describes the feedback from one part to the other. The following coupled differential equations governs the dynamics:

8 $$\begin{array}{rcl}\frac{dS}{dt} & = & {a}_{h}s+k{a}_{h}y-\beta ^{\prime} Sz+p{\gamma }_{2}z-{\delta }_{2}S-\mu S\\ \frac{dy}{dt} & = & \beta sy-{\gamma }_{1}y-k{a}_{h}y-\mu y\\ \frac{dz}{dt} & = & \beta ^{\prime} (s+S)z-{\gamma }_{2}z-\mu z\\ \frac{dh}{dt} & = & ({r}_{h}h+{r}_{l})-{\delta }_{1}h-\rho {c}_{z}z\end{array}$$

where $${a}_{h}=(\frac{\tilde{a}-\hat{a}}{{h}_{o}})h+\hat{a}$$, and *s* + *S* + *y* + *z* = 1.

For simplicity, we assume that *r*_*h*_ and *r*_*l*_ are linear function of *y* and *z* as follows: ${r}_{h}(y,z) \sim {r}_{h}^{c}\mathrm{(1}-y-z)$, ${r}_{l}(y,z) \sim {r}_{l}^{c}\mathrm{(1}-y-z)$. In the integrated model, we also assume that there is capital depreciation due to treatment of infected individuals by resistant strain. The term *ρc*_*z*_*z* denotes the depreciation, where *ρ* is probability of maximum individual treated, and *c*_*z*_ is per capita cost of treatment of severe infections. The variables and parameters descriptions are given in the Table 1 and in Supplementary Information.

#### Equilibrium solutions

The dynamics of the system with either of strains (*y* = 0 or *z* = 0) is much simpler than that of complete model given by (8). Here, we aim to discuss the effect of antibiotic consumption on the dynamics of both sensitive and resistant strains in the population, and hence, mainly consider the existence of endemic equilibrium when both pathogens exist in the system. Discussion of the endemic equilibrium provides us a framework to address interesting issues such as invasiveness of resistance in the community, and also defines parametric regime related to antibiotic use when resistant strains will come to dominate over sensitive strains, once present in the population.

#### Endemic equilibrium

It is the condition where the commensals are not entirely eliminated from the community. So, we have the susceptible individuals with and without antibiotic consumptions, and the infected individuals either with sensitive or the resistant strain. The endemic equilibrium is given by

$$\begin{array}{c}{P}^{\ast }({s}^{\ast },{S}^{\ast },{y}^{\ast },{z}^{\ast },{h}^{\ast }):(\frac{1}{{ {\mathcal R} }_{o}}+\frac{k{a}_{h}}{\beta },1-{y}^{\ast }-{s}^{\ast }\\ \,\,-{z}^{\ast },\frac{\mu +{\gamma }_{2}\mathrm{(1}-p)+\frac{{\delta }_{2}+{\gamma }_{2}(p-\mathrm{1)}}{{ {\mathcal R} }_{o}^{\text{'}}}-({a}_{h}+{\delta }_{2}+{ {\mathcal R} }_{o}^{\text{'}}\mu +{\gamma }_{2}({ {\mathcal R} }_{o}^{\text{'}}-\mathrm{1))}{s}^{\ast }}{k{a}_{h}+\mu +{\gamma }_{2}\mathrm{(1}-p)-(\mu +{\gamma }_{2}){ {\mathcal R} }_{o}^{\text{'}}{s}^{\ast }},\\ \,\,1-{y}^{\ast }-\frac{1}{{ {\mathcal R} }_{o}^{\text{'}}},\frac{{r}_{l}^{c}-\rho {c}_{z}{z}^{\ast }{ {\mathcal R} }_{0^{\prime} }}{{\delta }_{1}{ {\mathcal R} }_{o}^{\text{'}}-{r}_{h}^{c}})\end{array}$$

#### Conditions for existence of endemic equilibrium

1. Sensitive strain: $${y}^{\ast } > 0$$                                $${\rm{i}}{\rm{f}}\,\,{{\mathscr{R}}}_{0} > {{\mathscr{R}}}_{s}^{{\rm{^{\prime} }}}({a}_{h}):\,=\frac{{{\mathscr{R}}}_{0}^{{\rm{^{\prime} }}}({a}_{h}+{{\mathscr{R}}}_{0}^{{\rm{^{\prime} }}}(\mu +{\gamma }_{2})+{\delta }_{2})[1+\frac{k{a}_{h}}{\mu +{\gamma }_{1}}]}{\mu {{\mathscr{R}}}_{0}^{{\rm{^{\prime} }}}+{\delta }_{2}}$$
  The equilibrium density of sensitive strains y* is an decreasing function of antibiotic use *a*_*h*_. When *a*_*h*_ increases the threshold ${{\mathscr{R}}}_{s}^{{\rm{^{\prime} }}}({a}_{h})$ also increases. This condition explains that the basic reproduction rate of sensitive strain should be greater than the threshold value $({{\mathscr{R}}}_{s}^{{\rm{^{\prime} }}}({a}_{h}))$ to persist in the population in presence of resistant strain. Solving the inequality, the critical volume of antibiotic consumption to remove sensitive strain is given by
  9 $${a}_{h}^{c}=\sqrt{\frac{1}{4}{(B-\frac{1}{C})}^{2}+\frac{{ {\mathcal R} }_{0}A^{\prime} }{C}}-\frac{1}{2}(B+\frac{1}{C}),$$
  where *A*, *A*′, *B*, *C* are given by $A=\mu {{\mathscr{R}}}_{o}^{{\rm{^{\prime} }}}+{\delta }_{2}$, $B={{\mathscr{R}}}_{o}^{{\rm{^{\prime} }}}(\mu +{\gamma }_{2})+{\delta }_{2}$, $C=\frac{k{a}_{h}}{\mu +{\gamma }_{1}}$ and ${A}^{^{\prime} }=\frac{A}{{{\mathscr{R}}}_{o}^{{\rm{^{\prime} }}}}$. The detailed derivation of the threshold are given in the supplementary information.
2. Resistant strain: $${z}^{\ast } > 0\,\,if\,\,{ {\mathcal R} }_{0}^{^{\prime} } > { {\mathcal R} }_{r}^{^{\prime} }({a}_{h}):\,=\frac{1}{1-{y}^{\ast }({a}_{h})}$$
  For the resistance strain to invade, basic reproduction rate of resistance strain must be greater than the threshold value ${ {\mathcal R} }_{r}^{^{\prime} }({a}_{h})$, which is a decreasing function of *a*_*h*_.
3. Coexistence: $${ {\mathcal R} }_{co}^{1}({a}_{h}):=(1+\frac{k{a}_{h}}{\mu +{\gamma }_{1}}) < \frac{{ {\mathcal R} }_{0}}{{ {\mathcal R} }_{0}^{^{\prime} }} < { {\mathcal R} }_{co}^{2}({a}_{h}):\,=\frac{1}{{\delta }_{2}}(1+\frac{k{a}_{h}}{\mu +{\gamma }_{1}})({a}_{h}+{\delta }_{2}-{\gamma }_{2})$$

Condition (3) provides a parametric regime where both sensitive and resistant strains coexist in the system. While ${ {\mathcal R} }_{co}^{1}$ determines the density of susceptible population who are using antibiotics, the ${ {\mathcal R} }_{co}^{2}$ gives a threshold when the sensitive strain infects all individuals in the population. Both thresholds are increasing functions of *a*_*h*_, and thus, the entire population will be infected by the resistant strain *z*(*t*) at higher *a*_*h*_.

## Results

Conditions (1–3) discuss pathogen biology such as invasiveness and dominance of one strain on the other under different parameters such as rate (*a*_*h*_) and duration (1/*δ*_2_) of antibiotic consumption, level of awareness and education (*h*_0_). In absence of drug-resistant strain, the sensitive strain will be eradicated from the system if the antibiotic use reaches the threshold ${a}_{h}^{c}$ given by equation (9) with ${ {\mathcal R} }_{0}^{^{\prime} }=1$. An explicit expression is given in SI. Prolong use of antibiotics will also accelerate this eradication process, as the threshold ${ {\mathcal R} }_{s}^{^{\prime} }$ is not only a function of ${a}_{h}^{c}$, but also depends on *δ*_2_ i.e., the average duration of antibiotics consumption. However, in presence of drug-resistant strain, the eradication of sensitive commensal is much easier, as the value of the threshold ${a}_{h}^{c}$ decreases in presence of resistant strain (condition 1). Higher the values of ${ {\mathcal R} }_{0}^{^{\prime} }$, easier the eradication for sensitive strain. Figure S4 in SI shows that the threshold ${ {\mathcal R} }_{s}^{^{\prime} }$ is increasing as the basic reproduction rate of resistant strain ${ {\mathcal R} }_{0}^{^{\prime} }$ and antibiotic use *a*_*h*_ is increasing. Thus, it will be easier to eradicate sensitive strain with high volume of antibiotic use in presence of resistant strain.

Nonetheless, invasion of drug-resistant strain in a community with prior colonized by sensitive strain requires its basic reproduction rate is greater than ${ {\mathcal R} }_{r}^{^{\prime} }$ (condition 2), which decreases as the density of sensitive strain y* decreases in the population. As ${ {\mathcal R} }_{r}^{^{\prime} } > 1$, this signifies that colonization by drug-sensitive strain in the community can inhibit invasion by drug-resistant strain provided that the level of antibiotic consumption remains below a critical threshold ${a}_{h}^{c}$, and thus antibiotic consumption may escalate the invasion process of the resistant strain. This shows an instance, where prior colonization of one form of commensal can protect the community from the other one.

If drug-resistant strains have a transmission fitness advantage (which may be induced by antibiotic consumption), then its population will grow and system will evolve towards coexistence. The relative fitness of both strains determines the span of coexistence and it also depends on the niche created by antibiotic consumption rate *a*_*h*_ in population (condition 3). Increasing antibiotic consumption rate *a*_*h*_ increases the range by extending ${ {\mathcal R} }_{co}^{1}$ and ${ {\mathcal R} }_{co}^{2}$, but eventually ${ {\mathcal R} }_{0}^{^{\prime} }$ becomes greater than ${ {\mathcal R} }_{0}/{ {\mathcal R} }_{co}^{1}$, and all individual gets infected by the drug-resistant strain. Figure S5 in SI shows the long-term dynamics of two strains under different values of relative fitness (${ {\mathcal R} }_{0}^{^{\prime} }/{ {\mathcal R} }_{0}$) and volume of antibiotic use (obtained using *h*_0_, see Fig. S3 for refertence). The resistant population grows and sensitive population decreases as the relative fitness of resistant commensal increases, but the growth is much faster when the rate of antibiotic consumption increases. At the higher relative fitness and higher values of antibiotic use, only the resistant strain persist, wiping out the sensitive strain from the community.

In the numerical simulation, we mostly focus on the transient dynamics of the model system to see how the integrated model (8) connecting three domains of human population, creates a reinforcing cycle to initiate the development of resistance in population. Figure S6 in SI shows a sample time series explaining how the population density of a highly prevalent commensal (${ {\mathcal R} }_{0}^{^{\prime} } \sim 9$) can successfully grow by intense antibiotic treatment within a community. The eventual growth of resistance in the population has a negative impact on the economic growth, leading to lower income and hence promotes more antibiotic consumption by self-medication, which in turn, helps the resistance strain to grow and multiply towards stabilization. This phenomenon explains how three components (Fig. 1) yield synergistic interactions forming a reinforcing cycle in developing drug-resistance in the community.

### Impact of economy and awareness on the development of resistance

To investigate how socio-economic growth factors can promote the drug-resistance in the population, we assume that the resistant strain is already circulating in the population. In our model framework, we also differentiate populations based on socio-economic growth characterized by two important parameters in our model: the initial value of the variable *h*, i.e. *h*(0), which indicates economic status of the population in the beginning, and *h*_0_ which signifies education and awareness-level of individuals in the population. For example, low *h*(0) represents a population with poor economic status, but lower *h*_0_ represents relatively higher education and public health awareness in the population (see Fig. S3 in SI). Thus, high *h*(0) together with lower *h*_0_ characterizes richer or developed countries, whereas lower *h*(0) together with high *h*_0_ represents underdeveloped countries.

**Figure 3:**
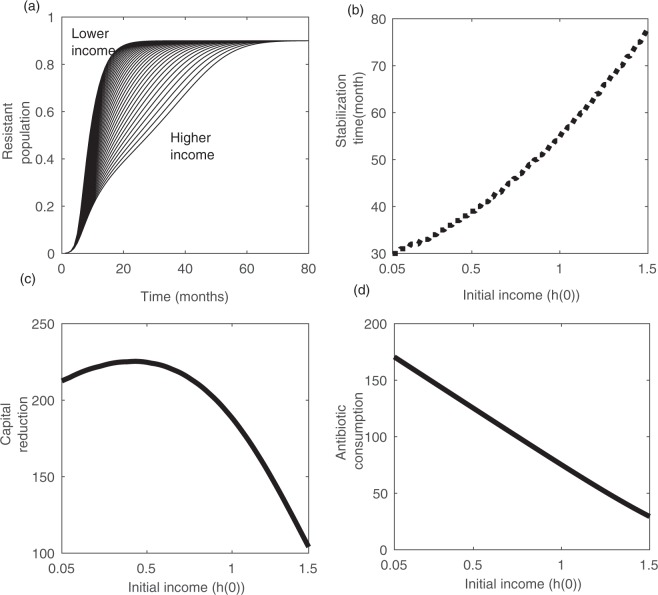
Impact of economic status (*h*(0)) on the development of the drug-resistance. (**a**) Time series of resistant strain for different values of *h*(0) - lower income drives early development of *z*(*t*). (**b**) Stabilization time, (**c**) income reduction, and (**d**) increase in antibiotic consumption when *h*(0) varies 0.05–1.5. Low income introduce more self medication, that drives early development of resistance, which in turn reduces more capital. Drug-resistance develops relatively quickly in low-income countries and so higher income reduction. For detail explanation, see the text.

Figure 3(a) shows the time series of resistant strain *z*(*t*) for different values of *h*(0). As this shows, the population with higher *h*(0) (representing relatively developed country), it takes 4 to 5 times longer duration to stabilize the resistant commensal compare to population with lower *h*(0). Moreover, this has equal effect on the economy and volume of antibiotic use by individuals in the population. This same scenario we have observed in many countries today – countries with lower-middle (LMIC) and upper-middle (UMIC) income (Fig. S1) accumulate more community-acquired resistance compared to developed countries^8,52–54^. To quantify the trajectory of development of resistance along different income status, we plot the time of stabilization of resistant strain *z*(*t*), and the area bounded by curves of *h*(*t*) and antibiotic use *a*_*h*_(*t*) for different values of *h*(0) (see Fig. S6 for reference). We observe that there is steady increase in the time to stabilization (Fig. 3(b)), and steady decrease in the volume of antibiotic consumption (Fig. 3(c)) as *h*(0) increases. However, the the amount capital reduction also decreases with higher economy (Fig. 3(d)), but there is plateau showing less changes in capital reduction for low values of *h*(0). Taken together, these results clearly explain how countries with lower economy can quickly develop a huge burden of community-acquired drug-resistance by increasing the volume of antibiotic misuse, and damaging the socio-economic growth.

We also experiment, in a similar way, the effect of awareness and education level on this dynamics, assuming that education and awareness can significantly reduce the self-medication and over-the-counter (OTC) antibiotic consumption. Figure S7(a) shows that population with lower education level (higher *h*_0_) accumulates the resistance much faster than population with higher education level (lower *h*_0_), though altogether the effect is less compare to economy *h*(0) (compare Fig. 3(a)). The trajectory of stabilization time (Fig. S7(b)) decreases with decrease in education level, and similarly the volume of antibiotic consumption (Fig. S7(c)) and capital reduction (Fig. S7(d)) increases as education and awareness level decreases. Thus, better economic conditions and better public health awareness may slow down the process of development of drug-resistant in population.

**Figure 4:**
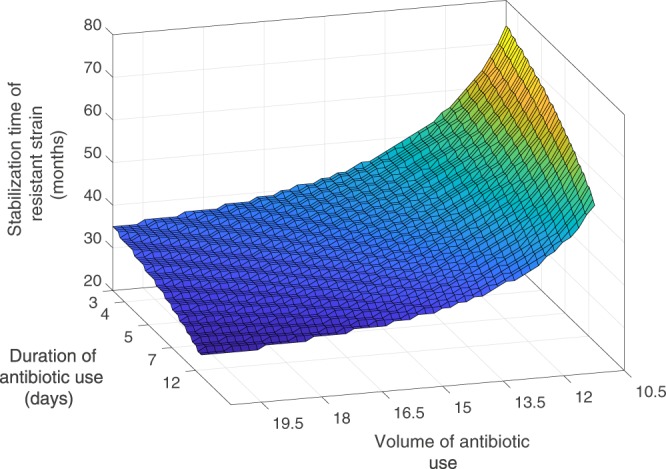
Stabilization time of emergence of resistant strain under different volume and duration of antibiotic consumption. The volume of antibiotic use corresponds to the area inside the triangle for each *h*_0_. When both volume and duration are high, the resistance develops very quickly, but volume of consumption has larger impact than the duration on the emergence process. For details, see the text.

To characterize the relation between development of drug-resistance and antibiotic consumption, we also plot the stabilization time of resistant strain with the volume and the duration of antibiotic use. As the Fig. 4 shows, stabilization of resistant strain varies significantly on the volume than the duration of consumption. It is four-fold higher time to stabilize when volume of antibiotic consumption is very low. However, the duration of consumption has greater impact when the volume of use is relatively lower. Hence, this indicates that public health policy-makers requires to be more concerned on the per capita antibiotic use rather than the period of use.

### Controlling the drug-resistance

**Figure 5:**
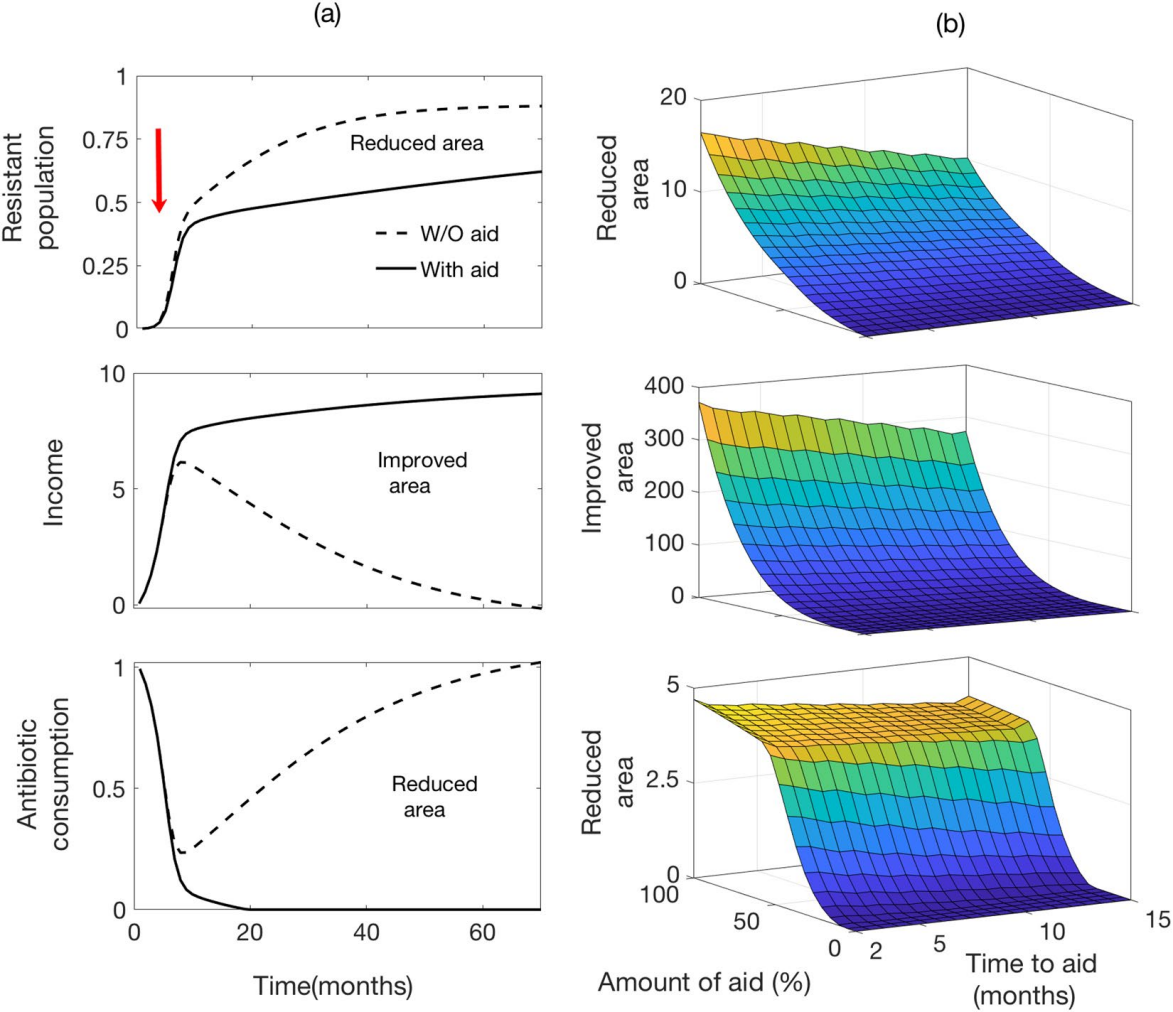
(**a**) Impact of government aid to control the drug-resistance. While the dotted curve depicts the dynamics of system before implementation of aid, the bold curve represents the effect after implementing the aid by reducing the cost of treatment *c*_*z*_. The original cost of treatment is assumed *c*_*z*_ = 12. The government provides aid at the 5^*th*^ month onward, then (i) curve of resistant population started declining, (ii) income curve rises up (iii) antibiotic consumption curve goes down. (**b**) Plot of area bounded by the dotted and bold curves in respective figures. Along with baseline values, other parameter values used for this simulation are ${R}_{o}=\mathrm{7,}\,{\delta }_{1}=\mathrm{0.11,}\,{\delta }_{2}=\mathrm{2,}\,{h}_{o}=\mathrm{7,}\,\hat{a}=\mathrm{2,}\,\tilde{a}=\mathrm{0.1,}\,{r}_{h}^{c}=\mathrm{0.9,}\,{r}_{l}^{c}=\mathrm{0.9,}\,\rho =\mathrm{0.07.}$ For details, see the text.

Our model demonstrates that community-acquired resistance may be viewed as self-reinforcing process via antibiotic consumptions, which incurs huge economic burden to the society. While the direct cost includes the prolonged hospitalization and treatment of patients, the indirect cost is due to the productivity losses from excess morbidity and mortality attributable to resistant infections. To control the situation, we require to slow down the process by perturbing the reinforcing cycle. One way to deal with this is that government should provide monetary aid such as reducing the treatment cost or providing free health service to the individuals infected by resistant strains. So, we experiment the effect of the monetary help on the dynamics of drug-resistance by reducing the per capita cost of treatment *c*_*z*_ in model equations (8). To replicate more realistic scenario, we also consider that the rate *ρ* of maximum infected individuals are treated is a decreasing function of *c*_*z*_ by

10 $$\rho ({c}_{z})={e}^{-m{c}_{z}},$$

that is, more people look for treatment when cost of treatment is low. Figure 5(a) shows the impact of government aid on the dynamics of resistance accumulation, capital reduction and antibiotic consumption over time. In the simulation, we reduce the cost of treatment *c*_*z*_ at the month five. The figure clearly shows that the population colonized with resistant starts declining, capital starts increasing as the capital curve rises up and hence, antibiotic consumption starts declining with the reduction in the cost of treatment. It is also observed that the reduction in the cost of treatment lift the low-income countries back on the track of economic advancement. Thus the amount of financial aid and timely initiative may help to decline the development of drug resistance in population.

However, the actual reduction in the burden of resistance depends on when and how much aid is allocated. To quantify this, we plot the area bounded by these two curves (before and after aid) in all three time series for different values of amount of aid and time-to-aid. As observed in the Fig. 5(b), the situation is much improved if the aid amount is higher and provided timely, especially, at the early stage of development of drug-resistant in the community. Subsidized treatment cost or 100% aid will maximize the reduction of resistance, but antibiotic mis(use) will drop to minimum at approximately 70% of aid. This analysis clearly underscores that agencies and policymakers need to be more proactive to combat the situation of antibiotic drug-resistance.

## Implication of Model Prediction And Empirical Pattern

Our theoretical framework explores the development of community-acquired antibiotic resistance as a consequence of antibiotic use, and discuss the implication of feedbacks in the dynamics from socioeconomic factors such as income, education and awareness. The model puts together population ecology of infection disease, antibiotic consumption and economic growth forming a vicious cycle, and predicts that the population acquires high burden of resistance in the long period of time that eventually leads the population to poverty. Empirical testing of such feedback loops or parameter estimation of such reinforcing dynamics among different components requires high-resolution data on transmission of resistance, antibiotic use, and impact of disease on the economic growth at both population- and individua-level. To our knowledge, there is rarely such data available in public repositories, and thus it becomes a serious challenge to test our theory. Instead, we allow our model to test for a more approachable goal of determining the plausibility, that is, can our model predicts a long term trajectory to reflect the empirical pattern observed in such population-level data?

**Figure 6:**
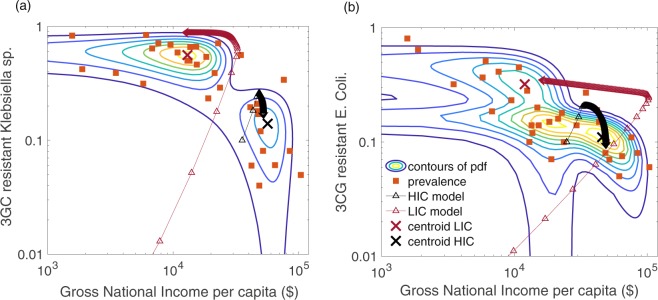
Resistance and gross National income percapita (GNIP). (**a**) Clusters of Klebsiella sp. data and model estimation. (**b**) Clusters of *E. Coli* data and model estimation. Refer the Fig. S1. The black cross mark and curve represents the HIC and red cross mark and curve represents the LIC. For details, see the main text.

We consider the data presented in Fig. S1 of the resistant prevalence of *Klebsiella sp*. and *E. coli* across different countries and the per capita gross national income (GNIP). As easily observed, the burden of resistance is negatively correlated with the gross income across countries, and this highly nonlinear negative correlation suggests that there is a possible interaction between these two components through some feedback loop such as one described above. To characterize empirical pattern in the data, we first perform a clustering analysis using Gaussian Mixture Model (GMM), which is commonly used technique in statistical data analysis for grouping data points or set of objects in such a way that data points in the same group are similar to one another in some sense and distinct from point in other groups. Detailed discussion on algorithm and results from the analysis are given in the supplementary information. The posterior probability obtained from the GMM indicates two components in spatial distribution of the dataset, where contours define the measure of dispersion of data points from the centroid of clusters under different confidence interval (Fig. 6). Results from the clustering analysis clearly illustrate that the burden of resistance is higher in low-income countries (LIC), but it is lower in relatively high-income countries (HIC).

We estimate model parameters such as the maximum and minimum antibiotic consumption level $\hat{a}$ and $\tilde{a}$, duration *δ*_2_, education and awareness level *h*_*o*_, and the cost *c*_*z*_ to treat per individual infected with the resistant strain. As seen in both resistant pathogens *Klebsiella sp*. and *E. coli*, the parameter estimates for LIC determine trajectory that approaches towards centroid of the clusters describing the higher burden of resistance, whereas the model solution with HIC parameter values converges towards the cluster of lower disease burden (Fig. 6). In fact, the basin of attraction for trajectories evolving towards centroid with higher disease burden can be seen as poverty trap. The parameter estimates are given in Tables S3 and S4 in the supplementary information. However, this model estimation supports the hypothesis that development of community-acquired drug-resistance can be seen as a self-reinforcing system that increases the overuse and misuse of antibiotics and hinders socioeconomic growth, eventually leading to poverty. While every country has its own economic constraints and epidemiological parameters behind the selection and emergence of resistance, this analysis suggests that the data are consistent with the theoretical framework considered here.

## Discussion

An important paramount question in recent upsurge of interest in global health is why burden of antibiotic drug-resistance follows very divergent trajectories in different parts of the world – it is converging to a controlled situation in some parts, but ever-growing in the other parts like in India, China, and Sub-Saharan Africa! A parallel predominant issue in global economy is also prevalent and increasing economic disparity between developed and developing countries – that the rich continues to grow richer, and the poor gets poorer. While there are many proposed explanations such as demographic^55^, political^4^, geographical^9^, and disease^45,56^ for this disparity from the theory of economics and other disciplines, there are only a few that attempt to understand this from the perspective of worldwide scenario of antibiotic drug-resistance burden^57^.

We have hypothesized and devised a model of drug-resistance amalgamating population ecology of infectious disease, socio-economic growth, and antibiotic (mis)use, that not only have the potential to demonstrate the divergence in drug-resistance profile among different populations, but also explains the ever-growing disparity in the global economy. The nonlinear interactions between income and health, and its consequence have been pointed out in many literatures, but how feedbacks between economy and health can generate self-reinforcing process by altering individuals behavior has been demonstrated in the present study. Empirical studies suggest that individuals from LMICs choose self-medication to avoid high treatment cost, and thus pointing towards a consistent increase in the gradient of antibiotic consumption. Our model also exhibits a similar pattern showing a negative relationship of drug-resistance with gross income or capital of populations. Low education and lack of awareness are other factors that promote self-medication. Currently, it has been observed that even the educated population of developing countries are indulged into this defective practice such as in India^19–21,58^. We have also shown using our model that low education and lack of awareness can also accelerate the emergence of resistant strains and toll a significant economic cost on the population. Although the general relationship between the burden of drug-resistance and economy of countries is interesting, it is more important to quantify the level of economic growth beyond which the antibiotic consumption increases rapidly. Such results are useful to control and policy interventions to manage antibiotic drug resistance. However, we need higher resolution data to quantify such thresholds in this complex process.

Theory from physics states that a self-reinforcing process – which is made of chain of complex events – continue in the direction of their momentum until an external factor intervenes and breaks the cycle. Our simple model also suggests that economic aid from government of countries or funding agencies can improve the present situation of drug resistance by perturbing the self-reinforcing feedback loop, although a late response may not be very effective. More the amount of aid and earlier the response, slower the development of resistance, and hence better the economic growth in population. Thus, providing monetary aid in the form of reduced treatment cost, inexpensive medicines and diagnostics are necessary for middle and low income countries, but that too before the establishment of resistant strain in community. Development aids can yield everlasting economic and health boon for the developing countries. Several international funding agencies such as GHIT, MOFA and MHLW in Japan, and Bill & Melinda Gates foundation in US are working to combat drug-resistance in developing countries. They are providing fund for development of new medicines, vaccines, and diagnostic tools to combat multi-drug resistant disease like HIV/AIDS, tuberculosis, malaria^59^, although the impact and effectiveness of foreign aid in developing countries depend on optimal allocation and coordination of the budget^60^.

While our theory provides a framework to explore such a complex system like antibiotic resistance, the model has been simplified in several aspects. There are scopes for further improvement. We examine the model in the absence of two biological mechanisms – mutation and plasmid transfer – which are important components for the selection and emergence of multi-drug resistant pathogens in population. Also, the transmission mechanisms of different pathogens are different – for example, malaria (vector-borne), HIV/AIDS (exposure to body fluid) and tuberculosis (Airborne). Our basic model may be improved and analyzed by integrating those specific characteristics of the pathogens. The model can also analyze the effect of the poor drug quality, which is another critical issue in selection and emergence of drug-resistant strains^61^. Here we consider linear production production function, which is a simpler approximation of actual constant-elasticity substitution (CES) function. Human behavior and social interactions are other issues that influence the antibiotic consumption in community^36^. Our study also motivates those who are readily curious to examine the interdependence of antibiotic resistance and economic by incorporating other socio-economic parameters like nutrition, hygienic level, lifestyle, living conditions, which serve as potential transmission factors of infection, along with migration, reproduction, and climate change that may expand the practicality of the model.

## Supplementary information

Supplementary Information

Supplementary Dataset 1

## Data Availability

Data is available as supplementary information.
