# Author Correction: Antibiotic drug-resistance as a complex system driven by socio-economic growth and antibiotic misuse

**DOI:** 10.1038/s41598-019-50846-1

**Published:** 2019-10-18

**Authors:** Bhawna Malik, Samit Bhattacharyya

**Affiliations:** Disease Modelling Lab, Department of Mathematics, School of Natural Sciences, Shiv Nadar University, Gautan Buddha Nagar, India

Correction to: *Scientific Reports* 10.1038/s41598-019-46078-y, published online 5 July 2019

In the Supplementary Information file originally published with this Article, the labelling of equations, figures and references in the text was omitted. These errors have been corrected in the Supplementary Information that now accompanies the Article.

