# Supplementary material for: Antibiotic drug-resistance as a complex system driven by socio-economic growth and antibiotic misuse
Source: Sci Rep. 2019 Jul 5;9:9788. doi: 10.1038/s41598-019-46078-y (PMC6611849; doi:10.1038/s41598-019-46078-y)
Supplement: Supplementary file 1 — Supplementary Information [file 41598_2019_46078_MOESM1_ESM.pdf]

Supplementary Material

Antibiotic drug resistance as complex system driven by socio-economic growth and antibiotic misuse

Bhawna Malik *[1] and Samit Bhattacharyya †[2]

[1,2]Disease Modelling Lab, Department of Mathematics, School of Natural Sciences, Shiv Nadar University, India

April 15, 2019

S1 Economic Growth Model

Neoclassical growth theory in economics describes the steady economic growth of a population under three driving forces namely, capital, labor and technology [1–3]. The relationship between the labor and capital determines the output. The theory states that advances in technology has sizable impact on an economy, and economic growth which is impossible without technological progress. A very well known production function is Constant-Elasiticity-of-Substitution (CES), which is one of the massively used function in economic analysis [1]. CES implies, any change in input factors, results in proportional change in the output. The function is algebraically shown as:

$$G(t) = \{r_h H(t)^\rho + r_l \ (L(t)^\rho)\}^{\frac{1}{\rho}} \tag{S1}$$

where $G(t)$ is the total output at time t, H is capital(income), L is labor supply. r_h and r_l are the factors sharing in total output from capital and labor respectively with $r_h + r_l = 1$. $\sigma = \frac{1}{(1-\rho)}$ is elasticity of substitution for capital and technology augmented labor. Depending on ρ, we can derive other form of the production function from equation (S1). For instance, 1) If $\rho = 1$, then linear production function, 2) If ρ approaches zero then it is Cobb-Douglas production function [4], 3) If ρ approaches infinity then Leontief production function [5].

*bm650@snu.edu.in

†Corresponding author: Tel: +91 120 3819100 (Extn. 136), Email: samit.b@snu.edu.in

Human capital which is another form of capital that is also sometimes fused into growth model. It represents the knowledge, education, competencies and health status [6, 7]. Health is regraded as human capital and also an input factor for generating different forms of capital. Unhealthy condition reduces the productivity and thus ability to devote in human capital [8].

Rate of change of capital depends on the saving and the amount depreciated. Repeatedly saving and investing a fragment of output for establishing new capital is the foot ground for capital accumulation and economic growth model [9]. According to the standard neo-classical growth model [1] rate of change of capital is given by:

$$\frac{dH}{dt} = G(t) - \delta_1 H(t) \tag{S2}$$

where, δ_1 is the rate of capital depreciation. Scaling the variables (i.e., $g \sim G/L$ and $h \sim H/L$) with per capita labor L, we have

$$\frac{dh}{dt} = g(t) - \delta_1 h(t), \tag{S3}$$

where $g = \{r_h h^\rho + r_l\}^{\frac{1}{\rho}}$. For simplicity, we assume $\rho = 1$ in our growth model.

S2 Two strain disease model: antibiotic resistance

We extend the classic Susceptible-Infected-Susceptible (SIS) framework to model the two strains - sensitive and resistant. We assume individual population are either colonized by sensitive strains or by drug resistant strains. We shall assume that drug resistant strain is already present circulating in the population. For simplicity, we consider that there is no mutation and plasmid transfer, and individuals can only be colonized by single strain at a time.

The population is divided into four compartments: $s(t)$ susceptible without antibiotics, $y(t)$ is population colonized by sensitive strain, $S(t)$ are susceptible who are taking antibiotics and $z(t)$ are individuals who are colonized with resistant strain and taking antibiotics.

μ is the mortality rate. β is the transmission rate of sensitive strain, and $\frac{1}{\gamma_1}$ is the average duration of colonization. β' is the transmission rate for resistant strain and $\frac{1}{\gamma_2}$ is the average duration of colonization. $\frac{1}{\delta_2}$ is the average duration of antibiotic consumption.

The equations for model are given by :

$$\frac{ds}{dt} = -(\beta y + \beta' z + a_h)s + \gamma_1 y + (1-p)\gamma_2 z + \delta_2 S - \mu s \tag{S4}$$

$$\frac{dS}{dt} = a_h s - \delta_2 S + k a_h y - \beta' S z + p\gamma_2 z - \mu S \tag{S5}$$

$$\frac{dy}{dt} = \beta s y - \gamma_1 y - k a_h y - \mu y \tag{S6}$$

$$\frac{dz}{dt} = \beta'(s+S)z - \gamma_2 z - \mu z \tag{S7}$$

$$s + S + y + z = 1 \tag{S8}$$

where, a_h is the antibiotic consumption is a function of income discussed in the main text. k is the proportion of individuals who gets recovered due to drug use. p is the fraction of individuals who are recovered, but still consume antibiotics.

S3 Calculation of equilibria and conditions

Using sensitive strain equation of integrated model in main text, we get $\frac{dy}{dt} = 0$
$\Rightarrow y^*(\beta s^* - \gamma_1 - k a_h - \mu) = 0 \Rightarrow s^* = \frac{1}{R_o} + \frac{k}{\beta}(mh^* + \hat{a})$

Using resistant strain equation, we get $\frac{dz}{dt} = 0$
$\Rightarrow z^*(\beta'(s^* + S^*) - \gamma_2 - \mu) = 0$. since $s + S + y + z = 1$ $z^* = 1 - y^* - \frac{1}{\mathcal{R}_o'}$

Using equation of capital or income, we get $\frac{dh}{dt} = 0$
$\Rightarrow g(h) = \delta_1 h(t) - \rho c_z z$. By substituting the value of r_h, r_l and z^*, we get $h^* = \frac{r_l^c - \rho c_z z^* \mathcal{R}_o'}{\delta_1 \mathcal{R}_o' - r_h^c}$

Now using equation of susceptible with antibiotics, $\frac{dS}{dt} = 0$

$\Rightarrow a_h s^* + k a_h y^* + p\gamma_2 z^* - S^*(\mu + \delta_2 + \beta' z^*) = 0$

Now using $S^* = 1 - y^* - s^* - z^*$, substituting the value of z^*, we have

$$y^* = \frac{\mu + \gamma_2(1-p) + \frac{\delta_2 + \gamma_2(p-1)}{\mathcal{R}_o'} - (a_h + \delta_2 + \mathcal{R}_o'\mu + \gamma_2(\mathcal{R}_o' - 1))s^*}{k a_h + \mu + \gamma_2(1-p) - (\mu + \gamma_2)\mathcal{R}_o' s^*}$$

Thus endemic equilibrium (s^*, y^*, z^*, h^*) is given by

$s^* = \frac{1}{\mathcal{R}_o} + \frac{k}{\beta}(mh^* + \hat{a})$

$y^* = \frac{\mu+\gamma_2(1-p)+\frac{\delta_2+\gamma_2(p-1)}{\mathcal{R}_o'}-(a_h+\delta_2+\mathcal{R}_o'\mu+\gamma_2(\mathcal{R}_o'-1))s^*}{ka_h+\mu+\gamma_2(1-p)-(\mu+\gamma_2)\mathcal{R}_o's^*}$

$z^* = 1 - y^* - \frac{1}{\mathcal{R}_o'}$

$h^* = \frac{r_l^c-\rho c_z z^* \mathcal{R}_o'}{\delta_1\mathcal{R}_o'-r_h^c}$

S3.1 Conditions for endemic equilibrium

1. $h^* = \frac{r_l^c-\rho c_z z^* \mathcal{R}_o'}{\delta_1\mathcal{R}_o'-r_h^c} > 0$

   if $\delta_1\mathcal{R}_o' - r_h^c > 0$ and $r_l^c - \rho c_z z^* \mathcal{R}_o' > 0 \Longrightarrow \frac{r_h^c}{\delta_1} < \mathcal{R}_o' < \frac{r_l^c}{\rho c_z z^*}$
   or

   if $\delta_1\mathcal{R}_o' - r_h^c < 0$ and $r_l^c - \rho c_z z^* \mathcal{R}_o' < 0 \Longrightarrow \frac{r_h^c}{\delta_1} > \mathcal{R}_o' > \frac{r_l^c}{\rho c_z z^*}$

2. $z^* > 0$ if $y^* < 1 - \frac{1}{\mathcal{R}_o'}$ i.e. if $\mathcal{R}_o' > \frac{1}{1-y^*(a_h)} = \mathcal{R}_r'(a_h)$

3. $y^* > 0$

   if $\mu + \gamma_2(1-p) + \frac{\delta_2+\gamma_2(p-1)}{\mathcal{R}_o'} - (a_h + \delta_2 + \mathcal{R}_o'\mu + \gamma_2(\mathcal{R}_o' - 1))s^* > 0$

   Discarding $\gamma_2(1-p) > 0$, we have $\frac{\mu\mathcal{R}_o'+[\delta_2+\gamma_2(p-1)]}{\mathcal{R}_o'(a_h+\delta_2+\mathcal{R}_o'(\mu+\gamma_2)-\gamma_2)} > \frac{1}{\mathcal{R}_o}[1 + \frac{ka_h}{\mu+\gamma_1}]$

   $\Rightarrow \mathcal{R}_o \geq \frac{\mathcal{R}_o'(a_h+\mathcal{R}_o'(\mu+\gamma_2)+(\delta_2-\gamma_2))[1+\frac{ka_h}{\mu+\gamma_1}]}{\mu\mathcal{R}_o'+\delta_2+\gamma_2(p-1)}$

   Discarding γ_2, $\mathcal{R}_o \geq \frac{\mathcal{R}_o'(a_h+\mathcal{R}_o'(\mu+\gamma_2)+\delta_2)[1+\frac{ka_h}{\mu+\gamma_1}]}{\mu\mathcal{R}_o'+\delta_2} =: \mathcal{R}_s'(a_h)$.

   In absence of resistant strain, the condition is reduced to $\mathcal{R}_o \geq \frac{(a_h+\mu+\delta_2)[1+\frac{ka_h}{\mu+\gamma_1}]}{\mu+\delta_2}$

4. $S^* > 0$ if $1 - y^* - s^* - z^* > 0 \Rightarrow s^* < \frac{1}{\mathcal{R}_o^{'}} \Rightarrow \frac{1}{\mathcal{R}_o^{'}} > \frac{1}{\mathcal{R}_o} + \frac{k}{\beta} a_h \Rightarrow \frac{R_o}{\mathcal{R}_o^{'}} < 1 + \frac{ka_h}{\mu+\gamma_2}$

5. $y^* < 1$ if $\frac{\mu+\gamma_2(1-p)+\frac{\delta_2+\gamma_2(p-1)}{\mathcal{R}_o^{'}}-(a_h+\delta_2+\beta^{'}-\gamma_2)s^*}{ka_h+\mu+\gamma_2(1-p)-\beta^{'}s^*} < 1$

   $\Rightarrow \mu + \gamma_2(1-p) + \frac{\delta_2+\gamma_2(p-1)}{\mathcal{R}_o^{'}} - (a_h + \delta_2 + \beta^{'} - \gamma_2)s^* < ka_h + \mu + \gamma_2(1-p) - \beta^{'} s^*$

   $\Rightarrow \frac{\delta_2+\gamma_2(p-1)}{\mathcal{R}_o^{'}} < ka_h + (a_h + \delta_2 - \gamma_2)s^*$

   Substitute $s^* = \frac{1}{\mathcal{R}_o}(1 + \frac{ka_h}{\beta})$, we have $\frac{\delta_2+\gamma_2(p-1)}{\mathcal{R}_o^{'}} < ka_h + \frac{1}{\mathcal{R}_o}(a_h + \delta_2 - \gamma_2)(1 + \frac{ka_h}{\beta})$

   By discarding $\gamma_2(p-1)$ and ka_h, we have $\frac{\mathcal{R}_o}{\mathcal{R}_o^{'}} < \frac{1}{\delta_2}(1 + \frac{ka_h}{\beta})(a_h + \delta_2 - \gamma_2)$. Using above, we have condition for co-existence

$$(1 + \frac{ka_h}{\mu + \gamma_1}) < \frac{\mathcal{R}_o}{\mathcal{R}_o^{'}} < \frac{1}{\delta_2}(1 + \frac{ka_h}{\beta})(a_h + \delta_2 - \gamma_2) \quad \text{(S9)}$$

S3.2 Threshold for a_h

$y^* = 0 \Rightarrow \mathcal{R}_o = \frac{(a_h+B)(1+C)}{A^{'}}$, where $A = \mu\mathcal{R}_o^{'} + \delta_2$, $B = \mathcal{R}_o^{'}(\mu + \gamma_2) + \delta_2$, $C = \frac{ka_h}{\mu+\gamma_1}$ and $A^{'} = \frac{A}{\mathcal{R}_o^{'}}$

Solving the equation for a_h and considering the positive root, we have $a_h = \frac{\sqrt{(BC-1)^2+4C\mathcal{R}_oA^{'}}-(BC+1)}{2C}$

S4 Clustering analysis of the data and model estimates

We use Gaussian Mixture Model (GMM) to perform cluster analysis of the GNIP-prevalence datasets (Figure S1). It is a commonly used technique in statistical data analysis for grouping data points or set of objects in such a way that data points in the same group are similar to one another in some sense and distinct from point in other groups. The gaussian mixture implements the expectation-maximization(EM) algorithm for fitting mixture-of-Gaussian models.

Based on Low and High per-capita income profile, we consider our GMM made of two multivariate normal density components, where each component has 2 dimensional mean, 2-by-2 covariance matrix, and a mixing proportion that decides the fraction of the population contributes to the respective component. We use MATLAB to run the algorithm and fit GMMs to the respective dataset. This iterative algorithm computes posterior probabilities for component membership for each observation calculating the component

means, covariance matrices, and mixing proportion by estimating maximum likelihood, using the component-membership posterior probabilities as weights. The results are listed in the table S1 and S2.

We use the centroid mean obtained from cluster analysis to estimate the dynamic model parameters. The income per-capita is scaled accordingly. We only estimate parameters that are related to the antibiotic consumption, and economic growth of the population. Other parameters are considered as base values.

Table S1: GMM cluster analysis for Klebsiella sp.

	Component1		Component2	
	GNIP	Prevalence	GNIP	Prevalence
Mean	12983.7345	55.6690	56481.9465	13.9335
Covariance (10^7(1)&10^8(2))	6.7606<br>-0.0028	-0.0028<br><0.00001	4.1512<br>-0.0004	-0.0004<br><0.00001
Component proportion	0.6036		0.3964	
AIC	1005.057237			
BIC	1021.180332			

Table S2: GMM cluster analysis for E.coli

	Component1		Component2	
	GNIP	Prevalence	GNIP	Prevalence
Mean	11980.4812	32.22717	45760.9158	11.44985
Covariance(10^7(1)&10^8(2))	7.19994<br>-0.0103	-0.0103<br><0.0001	6.5632<br>-0.0007	-0.0007<br><0.0001
Component proportion	0.4766		0.5234	
AIC	1002.136103			
BIC	1018.597686			

Table S3: Model estimate for Klebsiella sp.

Parameters	HIC	LIC
$\hat{a}$	1	3
$\tilde{a}$	0.01	0.1
c_z	2	0.2
δ_2	8	3
h_o	8	15
ρ	0.11	0.05
$\mathcal{R}_o$	7	7
$\mathcal{R}_o^{'}$	9	9
$h(0)$	5	0.5

Table S4: Model estimate for E.coli

Parameters	HIC	LIC
$\hat{a}$	1	3
$\tilde{a}$	0.01	0.1
c_z	2	0.2
δ_2	8	3
h_o	8	15
ρ	0.11	0.05
$\mathcal{R}_o$	7	7
$\mathcal{R}_o^{'}$	9.3	11
$h(0)$	3.5	0.5

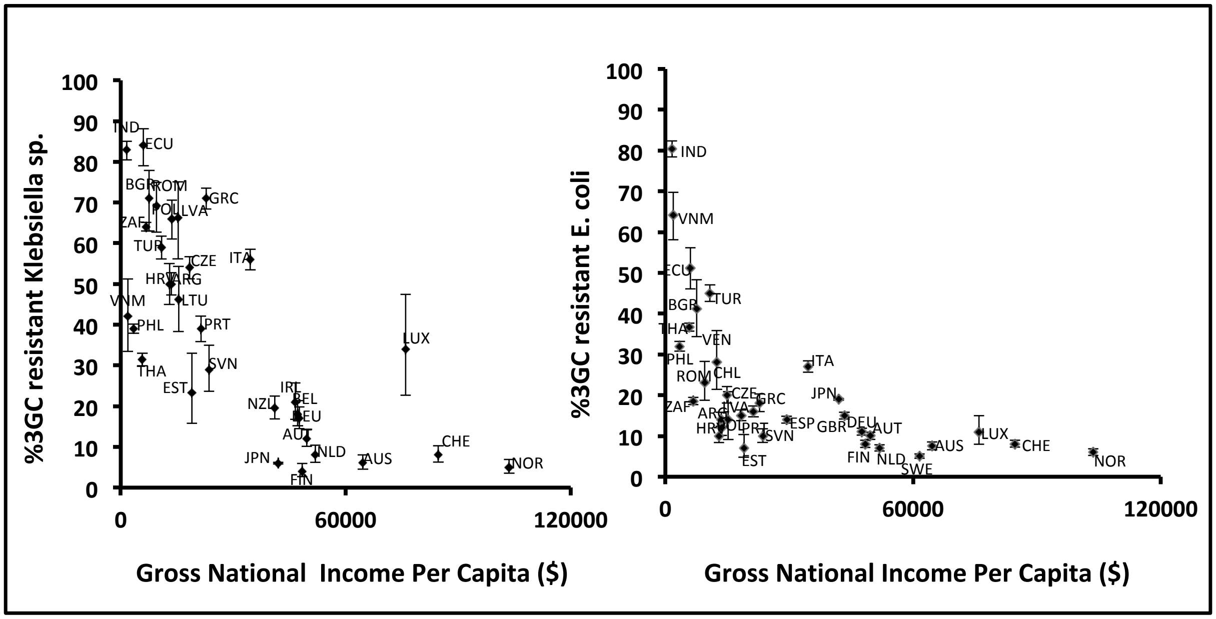

Figure S1: Prevalence of third-generation cephalosporin-resistant (3GCR) Klebsiella sp. (upper panel) and Escherichia coli (lower panel) by Gross National Income (GNI) per capita. The trend line for both HIC and MIC show negative relationship with GNI per capita. For details, see the text. Data have been adapted from [10] and the World Bank group.

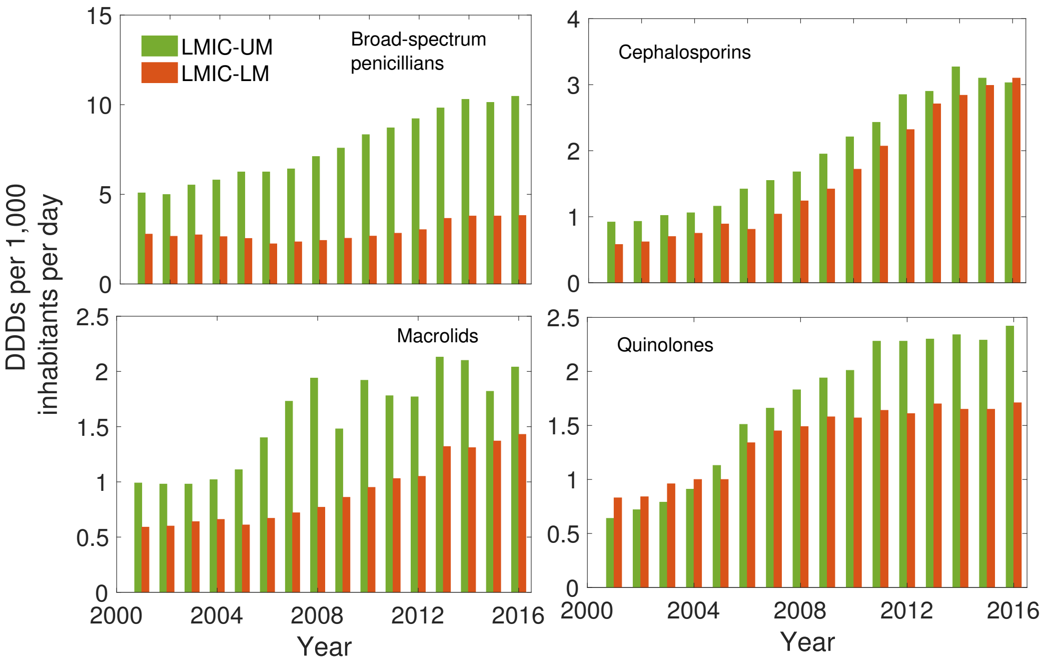

Figure S2: Different antibiotic consumption rate for Low middle income countries-lower middle($LMIC\text{-}LM$) and upper middle($LMIC\text{-}UM$) for the year 2000-2015. Data have been adapted from [11].

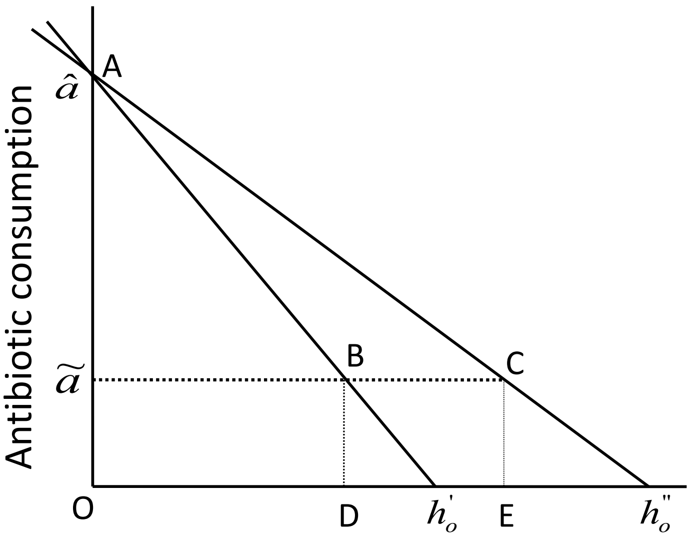

Figure S3: The Figure shows that antibiotic consumption is a linear function of income. Higher h_o determines low level of awareness, education. Triangle $\tilde{a}$AB shows the area of antibiotic consumption. When h_o increases the area of consumption also increases given by triangle $\tilde{a}$AC.

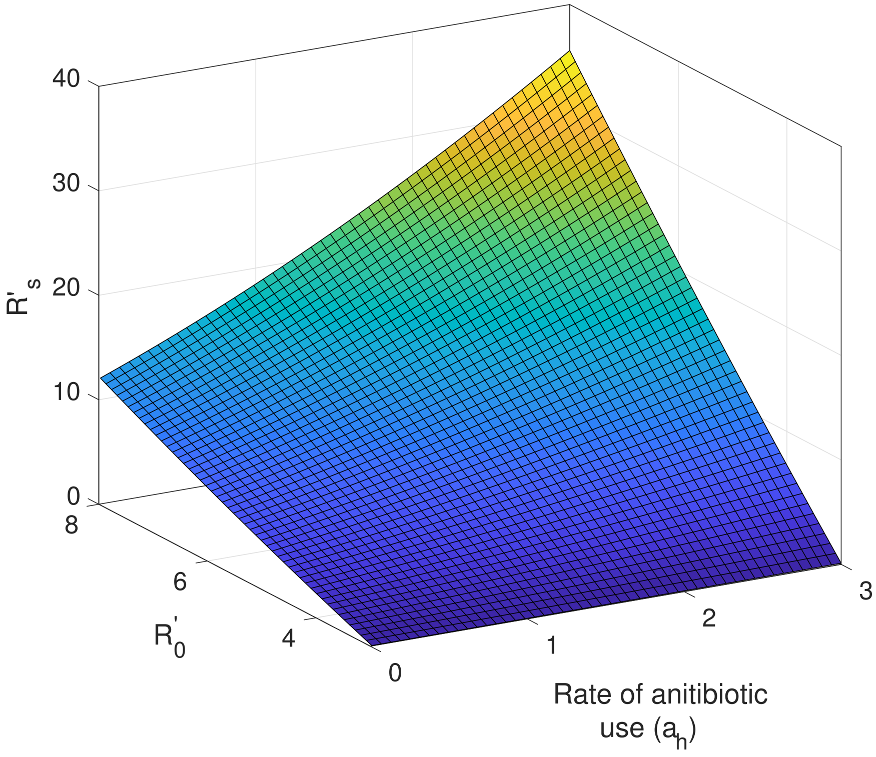

Figure S4: The threshold $\mathcal{R}_s'$ as function of $\mathcal{R}_0'$ and a_h. The threshold is increasing as the basic reproduction rate of sensitive strain and antibiotic use is increasing.

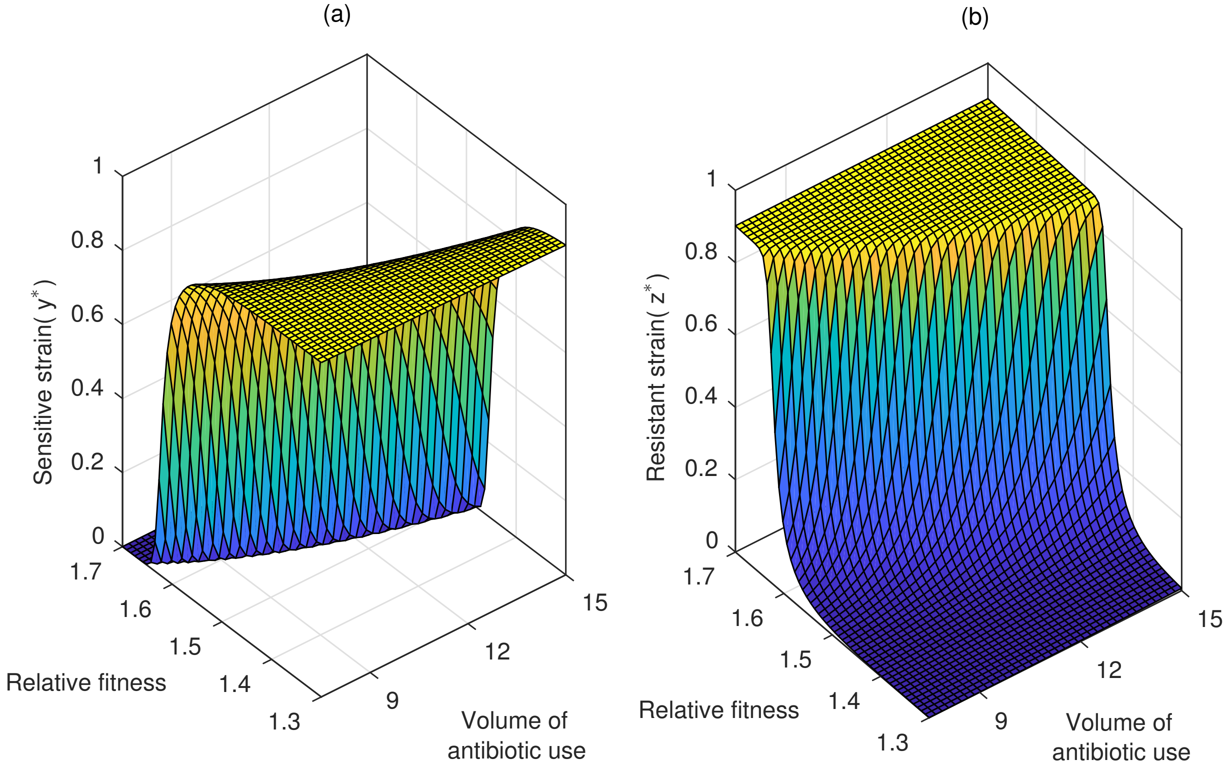

Figure S5: Prevalence of sensitive and resistant strains at different relative fitness of both strains $\mathcal{R}_0'/\mathcal{R}_0$ and volume of antibiotic use obtained from the Figure S3.

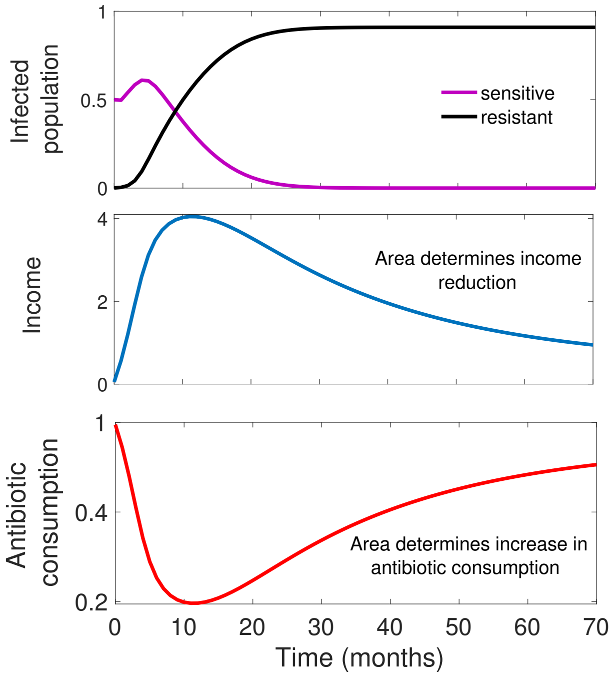

Figure S6: Time series plot of sensitive (y) and resistant (z) strains. The middle panel exhibit the evolution of income or capital h in the population, and lower panel shows antibiotic consumption. The area bounded by the tails of income curve determine the total reduction of income or capital due to emergence of resistance in the population. Similarly, area pointed in the lower panel exhibits the increase in total antibiotic consumption in population.

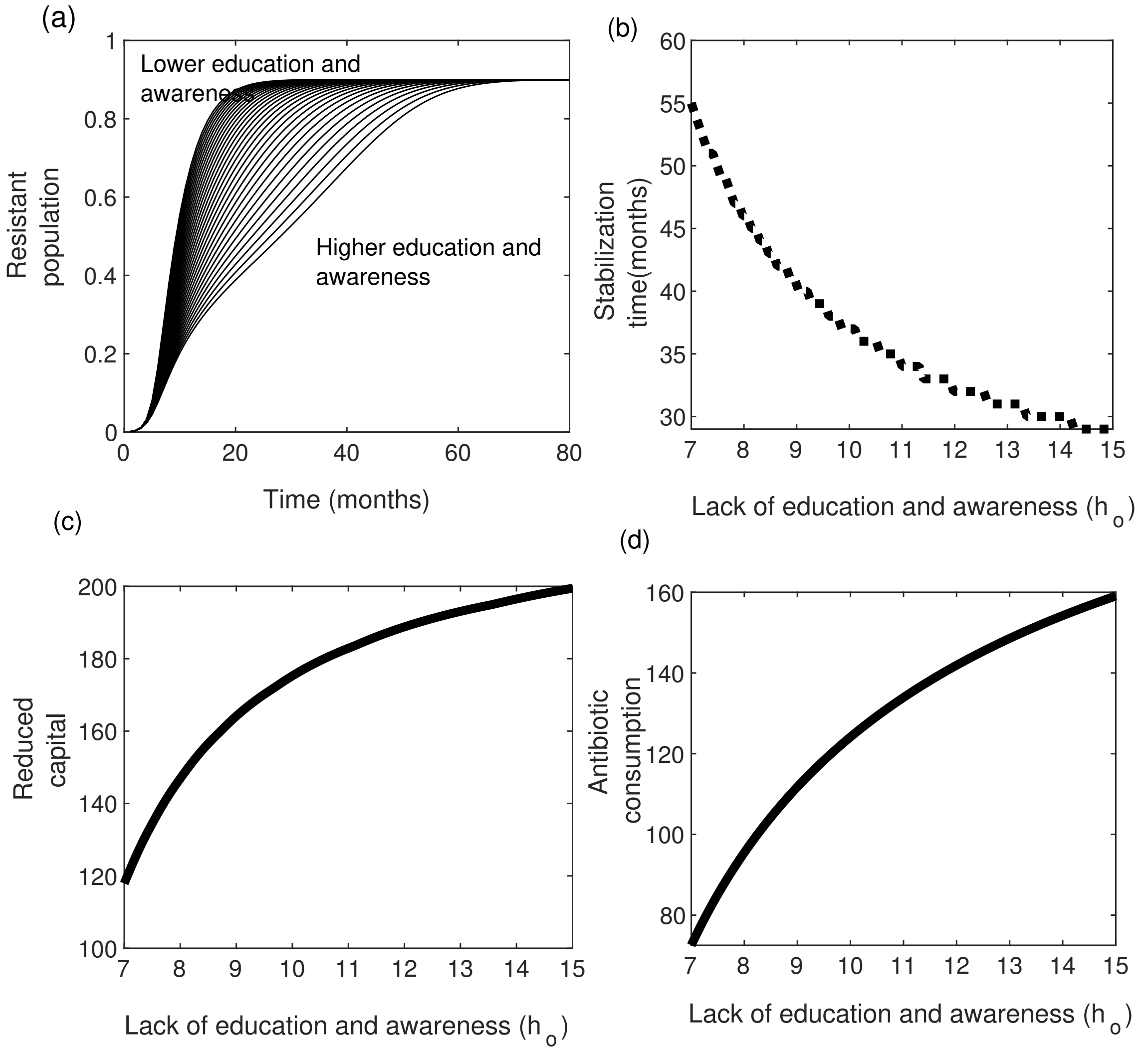

Figure S7: Impact of education and awareness (h_o) on the stabilization of resistance strain in the population. Figure (a) represents time series of resistant strain for different values of h_o. Low value of h_o signifies countries with higher education and awareness, while high values is for countries with less awareness. (b) stabilization time, (c) reduces capital, and (d) antibiotic consumption under different h_o. For detail explanation, see the text.

References

[1] R. M. Solow. A contribution to the theory of economic growth. *QJE*, 70(1):65–94, 1956.

[2] R. M. Anderson and R. M. May. *Infectious diseases of humans: dynamics and control.* Oxford university press, 1992.

[3] D. T. Quah. Twin peaks: growth and convergence in models of distribution dynamics. *The Economic Journal*, pages 1045–1055, 1996.

[4] I. N. Okeke, A. Lamikanra, and R. Edelman. Socioeconomic and behavioral factors leading to acquired bacterial resistance to antibiotics in developing countries. *Emerging infectious diseases*, 5(1):18, 1999.

[5] WW Leontief. The structure of the american economy, 1919–1929 harvard university press. *Cambridge (new, enlarged edition, Oxford University Press, New York, 1951)*, 1941.

[6] J. Zhang, J. Zhang, and R. Lee. Rising longevity, education, savings, and growth. *J Dev Econ*, 70(1):83–101, 2003.

[7] P. Aghion, P. Howitt, and F. Murtin. The relationship between health and growth: when lucas meets nelson-phelps. Technical report, NBER, 2010.

[8] H. Bleakley. Health, human capital, and development. *Annu. Rev. Econ.*, 2(1):283–310, 2010.

[9] C.N. Ngonghala, M. M. Pluciński, M. B. Murray, P. E. Farmer, C. B. Barrett, D. C. Keenan, and M. H. Bonds. Poverty, disease, and the ecology of complex systems. *PLoS biology*, 12(4):e1001827, 2014.

[10] G. Alvarez-Uria, S. Gandra, and R. Laxminarayan. Poverty and prevalence of antimicrobial resistance in invasive isolates. *IJID*, 52:59–61, 2016.

[11] E. Y. Klein, T. P. V. Boeckel, E. M. Martinez, S. Pant, S. Gandra, S. A. Levin, H. Goossens, and R. Laxminarayan. Global increase and geographic convergence in antibiotic consumption between 2000 and 2015. *Proceedings of the National Academy of Sciences*, page 201717295, 2018.
