# Supplementary material for: Antibiotic drug-resistance as a complex system driven by socio-economic growth and antibiotic misuse
Source: Sci Rep. 2019 Jul 5;9:9788. doi: 10.1038/s41598-019-46078-y (PMC6611849; doi:10.1038/s41598-019-46078-y)
Supplement: Supplementary file 2 — Supplementary Dataset 1 [file 41598_2019_46078_MOESM2_ESM.pdf]

Supplementary Dataset

Title: **Antibiotic drug-resistance as a complex system driven by socio-economic growth and antibiotic misuse**

Authors: **Bhawna Malik and Samit Bhattacharyya**
Disease Modelling Lab, Department of Mathematics, School of Natural Sciences, Shiv Nadar University, India

Email: bm650@snu.edu.in
samit.b@snu.edu.in

KLEBSIELLA

		Country	abbrevation	GNIP	mean	dif lb	Diff ub
LMIC (996-3895)							
	1	India	IND	1570	82.882	2.4165	2.1731
	2	Ecuador	ECU	6090	84.0637	5.0364	4.0096
	3	Philippines	PHL	3500	39.0019	1.1073	1.1187
	4	Vietnam	VNM	1890	42.1053	8.6607	9.1753
	5	Thailand	THA	5780	31.4225	1.5621	1.6054
UMIC (3896-12055)		South Africa	ZAF	6800	64.0197	1.0635	1.0499
		Bulgaria	BGR	7620	71.0145	8.0572	6.9189
		Romania	ROM	9520	69.1589	6.4801	5.8043
		Turkey	TUR	10830	58.9897	2.8457	2.7868
		Croatia	HRV	12980	50	5.0283	5.0282
		Argentina	ARG	13480	50	2.7161	2.7161
		Poland	POL	13680	65.9574	4.9294	4.6067
		Latvia	LVA	15250	66.3044	10.1391	8.832
		Lithuania	LTU	15410	46.2069	7.9122	8.1079
HIC		Australia	AUS	64600	6.05227	1.51314	1.97514
		Austria	AUT	49600	12.0085	1.924	2.2329
		Belgium	BEL	47240	18.0135	2.8819	3.2929
		Estonia	EST	19010	23.3333	7.5351	9.7184
		Finland	FIN	48440	4	1.34384	1.98196
		Czech Republic	CZE	18350	53.9891	2.7265	2.703
		Ireland	IRL	46520	20.8861	4.1189	4.8182
		Italy	ITA	34580	56.0028	2.5755	2.5436
		Japan	JPN	42000	5.99964	0.17776	0.18283
		Netherlands	NLD	51860	8.07453	1.864	2.36117
		New Zealand	NZL	41070	19.5219	2.6734	2.9828
		Norway	NOR	103620	4.96124	1.42529	1.9586
		poRTUGUAL	PRT	21360	38.9682	3.1142	3.2068
		Slovenia	SVN	23580	28.9796	5.3215	5.9705
		Switzerland	CHE	84720	8.06223	1.78756	2.24087
		Luxembourg	LUX	75960	33.9623	11.2753	13.4429
		Germany	DEU	47590	16.9713	2.492	2.8216
		Greece	GRC	22810	71.0265	2.6216	2.4883

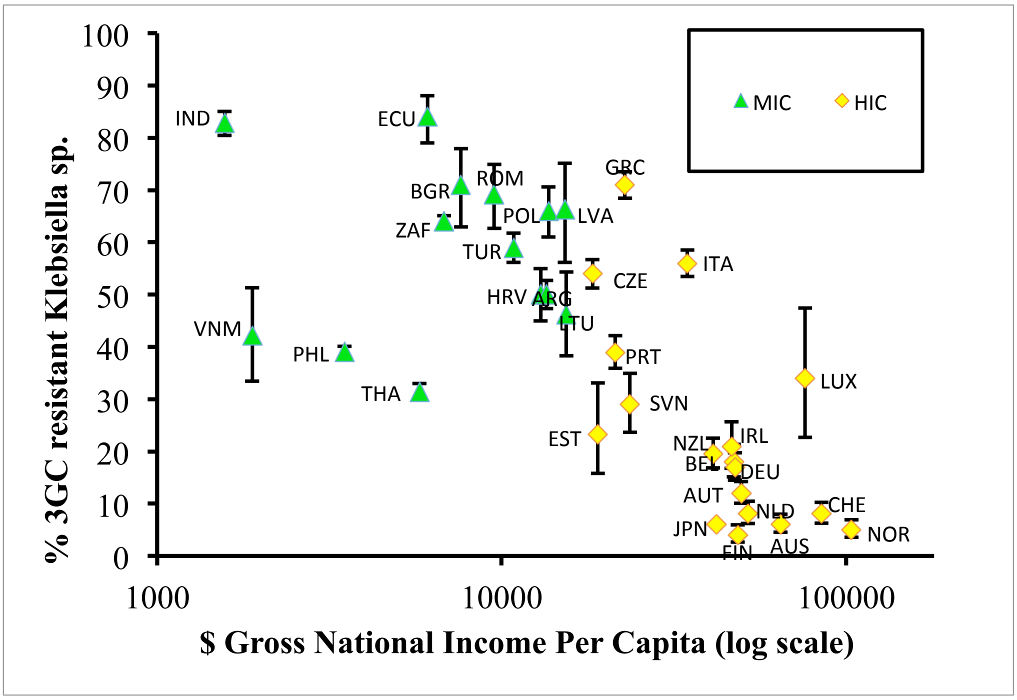
% 3GC resistant Klebsiella sp.
$ Gross National Income Per Capita (log scale)
MIC
HIC
100
90
80
70
60
50
40
30
20
10
0
1000
10000
100000
IND
ECU
BGR
ROM
POL
LVA
ZAF
TUR
HRV
ARG
LTU
VNM
PHL
THA
GRC
CZE
ITA
PRT
SVN
EST
LUX
NZL
IRL
BEL
DEU
AUT
NLD
CHE
JPN
FIN
AUS
NOR

			ecoli			
			GNIP	MEAN	Diff lb	diff ub
LMIC	India	IND	1570	80.4319	2.0644	1.9119
	Ecuador	ECU	6090	51.0695	5.0513	5.0296
	Philippines	PHL	3500	31.9941	1.2308	1.2564
	Vietnam	VNM	1890	64.0927	6.0092	5.5972
	Thailand	THA	5780	36.6756	0.9709	0.9818
UMIC	South Africa	ZAF	6800	18.5307	0.8417	0.8723
	Bulgaria	BGR	7620	41.1765	6.8072	7.1624
	Romania	ROM	9520	23.1544	4.4293	5.1125
	Turkey	TUR	10830	44.9843	2.0576	2.0748
	Venezuela	VEN	12500	28.0822	6.6556	7.7794
	Croatia	HRV	12980	10	1.67866	1.9731
	Argentina	ARG	13480	13.9799	1.6189	1.7926
	Poland	POL	13680	11.9691	1.8374	2.1184
	Chile	CHL	14910	19.9871	1.915	2.0633
	Latvia	LVA	15250	13.9706	4.84084	6.8203
HIC	Czech Republic	CZE	18350	14.9966	1.242	1.333
	Estonia	EST	19010	7.05882	2.26975	3.22928
	Portugal	PRT	21360	15.9821	1.339	1.4364
	Greece	GRC	22810	18.008	2.0273	2.2225
	Slovenia	SVN	23580	9.96732	1.55501	1.80548
	Spain	ESP	29390	13.9919	0.8595	0.9061
	Italy	ITA	34580	26.9925	1.3548	1.399
	Japan	JPN	42000	19.0001	0.217	0.2189
	Germany	DEU	47590	11.0028	0.8118	0.8679
	Finland	FIN	48440	8.01075	0.82964	0.91628
	Austria	AUT	49600	10.0091	0.8544	0.9246
	Netherlands	NLD	51860	7.00422	0.69228	0.76191
	Sweden	SWE	61570	5.00531	0.46992	0.51579
	Australia	AUS	64600	7.50507	0.89535	1.00558
	Luxembourg	LUX	75960	10.9635	3.04972	4.0335
	Switzerland	CHE	84720	8.00904	0.80306	0.88398
	Norway	NOR	103620	6.01235	0.78635	0.89604
	United Kingdom	GBR	43390	15.0015	0.842	0.8828

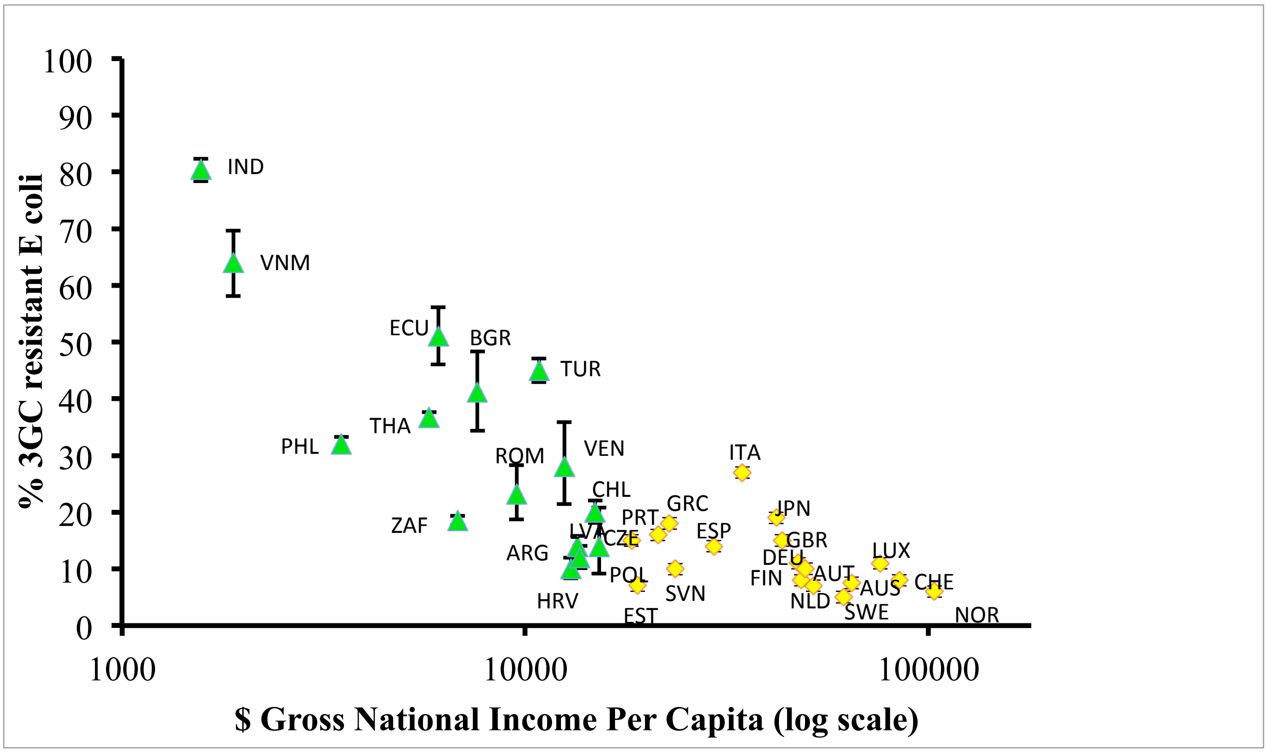

% 3GC resistant E coli
$ Gross National Income Per Capita (log scale)
100
90
80
70
60
50
40
30
20
10
0
1000
10000
100000
IND
VNM
ECU
BGR
TUR
THA
PHL
ROM
VEN
ITA
CHL
GRC
PRT
JPN
ZAF
LVA
CZE
ESP
GBR
ARG
DEU
LUX
POL
AUT
FIN
AUS
CHE
SVN
HRV
NLD
SWE
EST
NOR

Variables	Comment
mean	point estimate countrywise
lb	lower limit point estimate countrywise
ub	upper limit point estimate countrywise
abb	COUNTRY
gnipc1	Gross national income per capita
ihat	linear prediction
xblb	lower limit linear prediction
xbub	upper limit linear prediction

Broad-Spectrum Penicillins: DDDs per 1,000 inhabitants

year	LMIC-UM	LMIC-LM
2000	5.08	2.78
2001	4.99	2.66
2002	5.52	2.74
2003	5.80	2.64
2004	6.25	2.54
2005	6.25	2.24
2006	6.42	2.35
2007	7.11	2.43
2008	7.58	2.55
2009	8.33	2.67
2010	8.71	2.83
2011	9.22	3.03
2012	9.82	3.66
2013	10.30	3.79
2014	10.13	3.79
2015	10.47	3.82

Cephalosporins: DDDs per 1,000 inhabitants per day by

year	LMIC-UM	LMIC-LM
2000	0.92	0.58
2001	0.93	0.62
2002	1.02	0.70
2003	1.06	0.75
2004	1.16	0.89
2005	1.42	0.81
2006	1.55	1.04
2007	1.68	1.24
2008	1.95	1.42
2009	2.21	1.72
2010	2.43	2.07
2011	2.85	2.32
2012	2.90	2.71
2013	3.27	2.84
2014	3.10	2.99
2015	3.03	3.10

Macrolides: DDDs per 1,000 inhabitants per day by

year	LMIC-UM	LMIC-LM
2000	0.99	0.59
2001	0.98	0.60
2002	0.98	0.64
2003	1.02	0.66
2004	1.11	0.61
2005	1.40	0.67
2006	1.73	0.72
2007	1.94	0.77
2008	1.48	0.86
2009	1.92	0.95
2010	1.78	1.03
2011	1.77	1.05
2012	2.13	1.32
2013	2.10	1.31
2014	1.82	1.37
2015	2.04	1.43

Quinolones: DDDs per 1,000 inhabitants per day by

year	LMIC-UM	LMIC-LM
2000	0.64	0.83
2001	0.72	0.84
2002	0.79	0.96
2003	0.91	1.00
2004	1.13	1.00
2005	1.51	1.34
2006	1.66	1.45
2007	1.83	1.49
2008	1.94	1.58
2009	2.01	1.57
2010	2.28	1.64
2011	2.28	1.61
2012	2.30	1.70
2013	2.34	1.65
2014	2.29	1.65
2015	2.42	1.71
